# White Matter Microstructure in Adolescents and Young Adults With Non-Suicidal Self-Injury

**DOI:** 10.3389/fpsyt.2019.01019

**Published:** 2020-01-24

**Authors:** Melinda Westlund Schreiner, Bryon A. Mueller, Bonnie Klimes-Dougan, Erin D. Begnel, Mark Fiecas, Dawson Hill, Kelvin O. Lim, Kathryn R. Cullen

**Affiliations:** ^1^Department of Psychiatry, University of Utah, School of Medicine, Salt Lake City, UT, United States; ^2^Department of Psychiatry and Behavioral Sciences, University of Minnesota Medical School, Minneapolis, MN, United States; ^3^Department of Psychology, University of Minnesota College of Liberal Arts, Minneapolis, MN, United States; ^4^Division of Biostatistics, University of Minnesota School of Public Health, Minneapolis, MN, United States

**Keywords:** non-suicidal self-injury, neuroimaging, fractional anisotropy, uncinate fasciculus, cingulum, adolescents

## Abstract

**Background:**

Non-suicidal self-injury (NSSI) is a growing public health concern that commonly begins in adolescence, and can persist into young adulthood. A promising approach for advancing our understanding of NSSI in youth is to examine white matter microstructure using diffusion magnetic resonance imaging (dMRI).

**Method:**

The present study examined whole-brain group differences in structural connectivity (as measured by generalized fractional anisotropy [GFA]) between 28 female adolescents and young adults ages 13–21 years with NSSI and 22 age-matched healthy controls (HC). We also explored the association between clinical characteristics including NSSI severity and duration, impulsivity, emotion regulation and personality traits within the NSSI group and GFA of the uncinate fasciculus and cingulum.

**Results:**

Compared to the HC group, participants with NSSI had lower GFA in several white matter tracts, including the uncinate fasciculus, cingulum, bilateral superior and inferior longitudinal fasciculi, anterior thalamic radiation, callosal body, and corticospinal tract. When controlling for depressive symptoms, the NSSI group showed an association between NSSI duration (time since initiating NSSI behavior) and lower GFA in the left cingulum. Higher levels of attentional impulsivity were related to lower GFA in the left uncinate fasciculus within the NSSI group.

**Conclusions:**

We found evidence suggesting widespread white matter microstructure deficits in adolescents and young adults with NSSI versus HC. We also report inverse associations between white matter integrity and clinical characteristics (duration of NSSI and attentional impulsivity). These white matter microstructural deficits may represent a possible neurobiologically-based vulnerability to developing maladaptive coping mechanisms, such as NSSI. Additionally, results suggest that this white matter disorganization may either worsen with prolonged engagement in NSSI or predict persistent NSSI; thereby highlighting the importance of early intervention targeting this behavior.

## Introduction

Non-suicidal self-injury (NSSI), or the purposeful act of harming oneself without suicidal intent, commonly begins in adolescence and is associated with negative outcomes such as persistent psychopathology and suicide (1–3). Research examining the neurobiological correlates of NSSI is necessary to guide the development of biologically-informed interventions. Given that it is a particularly sensitive developmental period of significant neurobiological changes and the onset of NSSI, research may benefit from focusing on adolescence in particular (4–6).

The efficient transmission of neural signals depends in part on the organization and integrity of white matter fiber bundles and the structural characteristics of the myelin sheath that surrounds the body of an axon. These characteristics help to facilitate and constrain neuronal communication, thereby enhancing efficiency of neural functioning (7). Diffusion MRI (dMRI) is a brain imaging method that measures the diffusion of water molecules within the white matter of the brain. The dMRI metric of fractional anisotropy (FA) has traditionally been used used to estimate white matter organization. FA produces a value between zero and one, in which zero reflects complete isotropy (diffusion is not at all restricted or is restricted equally in all directions) and one reflects anisotropy (diffusion is confined to a particular direction). The assumption is that FA values reflect characteristics of white matter microstructure, such as myelination and directionality or coherence of white matter fiber bundles [see (8) for review]. In this case, higher FA values are typically interpreted as reflecting more optimal organization and integrity of white matter.

One challenge in dMRI research has been that when an MRI voxel captures multiple crossing fibers, the FA measurement will be artifactually low (9). To address this issue, a recent advance in dMRI research has been to use High Angular Resolution Diffusion Imaging (HARDI) acquisition, which allows for analysis strategies that may resolve multiple fiber directions in a voxel (10, 11). This can be accomplished by using spherical harmonization to calculate the Orientation Distribution Function (ODF), which is then used to estimate Generalized Fractional Anisotropy [GFA; (12)]. Similar to FA, higher GFA values indicate greater directionality of diffusion. GFA improves on the standard tensor model by being less susceptible to the effects of crossing or “kissing” white matter fibers (13, 14).

Only one study to date has used dMRI to examine white matter in patients with a history of self-injury (15). This study found that women with borderline personality disorder (BPD) and a history of self-injury had lower FA within the inferior frontal lobe compared to controls (15). However, study limitations included a small sample size (n = 9 BPD and 7 healthy controls), lack of clarity on whether the self-injury was suicidal or non-suicidal, use of FA as opposed to GFA, and lack of a dimensional approach to gain a deeper understanding of this biological finding.

Using advanced dMRI (HARDI) methods, this study examined GFA in adolescents and young adults with NSSI versus healthy controls. We hypothesized that the NSSI group would show lower GFA than controls. More specifically, given the association between difficulties in self-regulation and NSSI [see (16, 17) for review], we anticipated that this would include lower GFA within white matter tracts from neural circuits that are known to be involved in self-regulation, such as the uncinate fasciculus and cingulum (18, 19). Further, within the NSSI group, we examined clinical correlates of GFA within the uncinate fasciculus and cingulum. We predicted that lower GFA would be associated with greater NSSI severity and with greater difficulties in self-regulation.

## Method

### Participants

Data were used from a recently completed study at the University of Minnesota (Cullen: 1R21MH094558), which was approved by the University of Minnesota Institutional Review Board. Female healthy controls (HC) and participants with NSSI aged 13–21 years were recruited using primarily community postings, clinic referrals, and online advertisements around the Minneapolis/Saint Paul area. While the larger study was open to both males and females, only females were included for the present analyses as only one male participated. Inclusion criteria for the NSSI group included engaging in NSSI at least 4 times, with at least 1 episode occurring in the past month. Exclusion criteria for both groups was a history of bipolar, pervasive developmental or psychotic disorders, current pregnancy or breastfeeding, unstable medical illnesses, active suicidal intent, presence of MRI-incompatible features, a positive urine drug screen, and intelligence quotient (IQ) of less than 80 as measured by the Wechsler Abbreviated Scale of Intelligence [WASI; (20)]. Additional exclusion criteria for HC included any history of self-injurious behavior (suicidal or non-suicidal) and any current or past DSM-IV psychiatric diagnoses. Interested participants contacted the research team *via* email or phone, which was followed by a phone screen to assess for basic inclusion and exclusion criteria.

Participants who appeared eligible *via* the phone screen were invited to participate in the initial screening visit. Participants with NSSI were offered three different options for study participation: (1) MRI study only; (2) treatment study only; or (3) both MRI and treatment study (MRI conducted both pre- and post-treatment). The treatment offered was an open label pilot study for the dietary supplement N-acetylcysteine. Further description of this trial and its clinical results have been previously published (21). Participants in the HC group were only offered the option to participate in the MRI study. The present study includes data from participants who elected to complete the MRI-only study or the MRI and treatment study (using only the pre-treatment MRI data). Other neuroimaging data from this study (resting-state and task functional connectivity and psychophysiological interactions) have been published previously (22, 23). Once participants selected their desired study option, informed consent and assent (where applicable) were obtained.

### Measures

#### Clinical Assessment

Following informed consent and assent (as appropriate), all participants completed comprehensive diagnostic assessments, which were conducted by trained clinicians or graduate students or trainees under the supervision of a licensed psychologist or psychiatrist. Interviews were conducted separately with adolescents and parents, and included Kiddie Schedule for Affective Disorders and Schizophrenia-Present and Lifetime Version [K-SADS-PL; (24)] for participants under 18 years old and the Structured Clinical Interview for DSM-IV Axis I Disorders [SCID; (25, 26)] for participants 18 years old or older. For those under 18 years old, diagnoses were established *via* consensus between the adolescent and parent/guardian interviewers. Participants also completed the Beck Depression Inventory-II [BDI-II; (27)], which was used to control for depressive symptoms for within-NSSI group analyses.

##### Non-Suicidal Self-Injury

We assessed for NSSI using the self-report Inventory of Statements About Self-Injury [ISAS; (28)] and the clinician-administered Deliberate Self-Harm Inventory [DSHI; (29)]. These two measures were used to provide a consensus on frequency and type of self-injury as well as duration of NSSI for each participant in the NSSI group. Average weekly cutting episodes were calculated by taking the consensus of lifetime cutting episodes from the ISAS and DSHI and dividing them by the estimated number of weeks the participant engaged in NSSI. We focused on cutting episodes for these analyses because cutting was the primary method of NSSI among all the NSSI participants. We used winsorization to reassign outliers on this variable to three standard deviations above the mean. Duration of NSSI was calculated by subtracting the age participants reported first engaging in NSSI from their current age.

##### Self-Regulation Measures

Measures of self-regulation included the Difficulties in Emotion Regulation Scale [DERS; (30)] and the Barratt Impulsiveness Scale [BIS; (31)]. The DERS includes six subscales in addition to a total score: Awareness, Clarity, Goals, Impulse, Nonacceptance, and Strategies. The factor structure of the DERS was initially found among adults (30) and has been replicated among adolescents (32). The DERS has internal consistency that ranges from acceptable to high across factors in both adolescents (average α = .81) and adults (average α = .85). The BIS includes three subscales in addition to a total score: Attentional, Motor, and Non-planning. The BIS total score has been found to have high internal consistency, with α ranging from.79 in substance-abuse patients and.83 in general psychiatric patients (31). Total score and subscales from both the DERS and the BIS were used for analyses. In addition, we examined participants’ t-scores on the Self-Harm subscale within the Borderline clinical scale (BOR-S) from the Personality Assessment Inventory (PAI) or Personality Assessment Inventory-Adolescent (PAI-A) for those under 18 (33, 34). The BOR-S scale is a measure of self-destructive and impulsive behavior in general and includes questions regarding behaviors at high-risk for negative consequences. While the PAI and PAI-A differ in length (PAI has 344 items and PAI-A has 242 items), the conversion of raw scores to t-scores allows for the two measures to be comparable. Both measures have shown high test-retest reliability with correlations of.80 or higher for all subscales of the PAI and an average correlation of.78 for the PAI-A. Additionally, both measures have demonstrated high internal consistency for the scales, with a median α of.88 and average α of.80 for the PAI and PAI-A respectively (33, 34).

#### Neuroimaging Acquisition

Following the first visit (consent and diagnostic/clinical assessment), participants completed an MRI scan at the Center for Magnetic Resonance Research at the University of Minnesota using a Siemens 3T TIM Trio scanner and a 32-channel receive-only head coil. A pair of diffusion scans were acquired with identical parameters except with opposite phase encode directions (right to left and left to right) to estimate and correct for distortions. These scans were acquired using a multi-band EPI sequence with: 66 oblique axial slices; 2mm isotropic voxel; 128 volumes with non-colinear diffusion directions and 17 volumes without diffusion weighting; flip angle = 90°; FOV = 212mm; multiband factor = 3; b-value = 1,500 s/mm^2^; TR = 3,097 ms; TE = 90.2 ms.

### Statistical Analysis

#### Demographics and Clinical Data

Demographic and clinical data were analyzed using IBM SPSS Version 24 (35). Descriptive variables of interest included age, IQ, scores on clinical measures of psychopathology, and current psychiatric diagnoses and medications.

#### Diffusion MRI Preprocessing and Analysis

Image processing was performed using software from the FSL toolkit (https://fsl.fmrib.ox.ac.uk/fsl/fslwiki). The topup tool from FSL was performed on the pair of dMRI scans (i.e., the right to left phase encode pair) from each participant to estimate the susceptibility induced off-resonance field. Each scan pair was then concatenated using *fslmerge*. Eddy-current and susceptibility-induced distortion corrections were completed using the Gaussian Process approach applied by *eddy* in FSL (36). We used the Brain Extraction Tool (BET) in FSL to complete brain extraction on the resulting data. Custom built tools created in MATLAB, as developed by Aganj and colleagues (37) based on the method presented by Assemlal, Tschumperlé, and Brun (12), were used to calculate ODF and create GFA maps for each individual.

**Figure 1:**
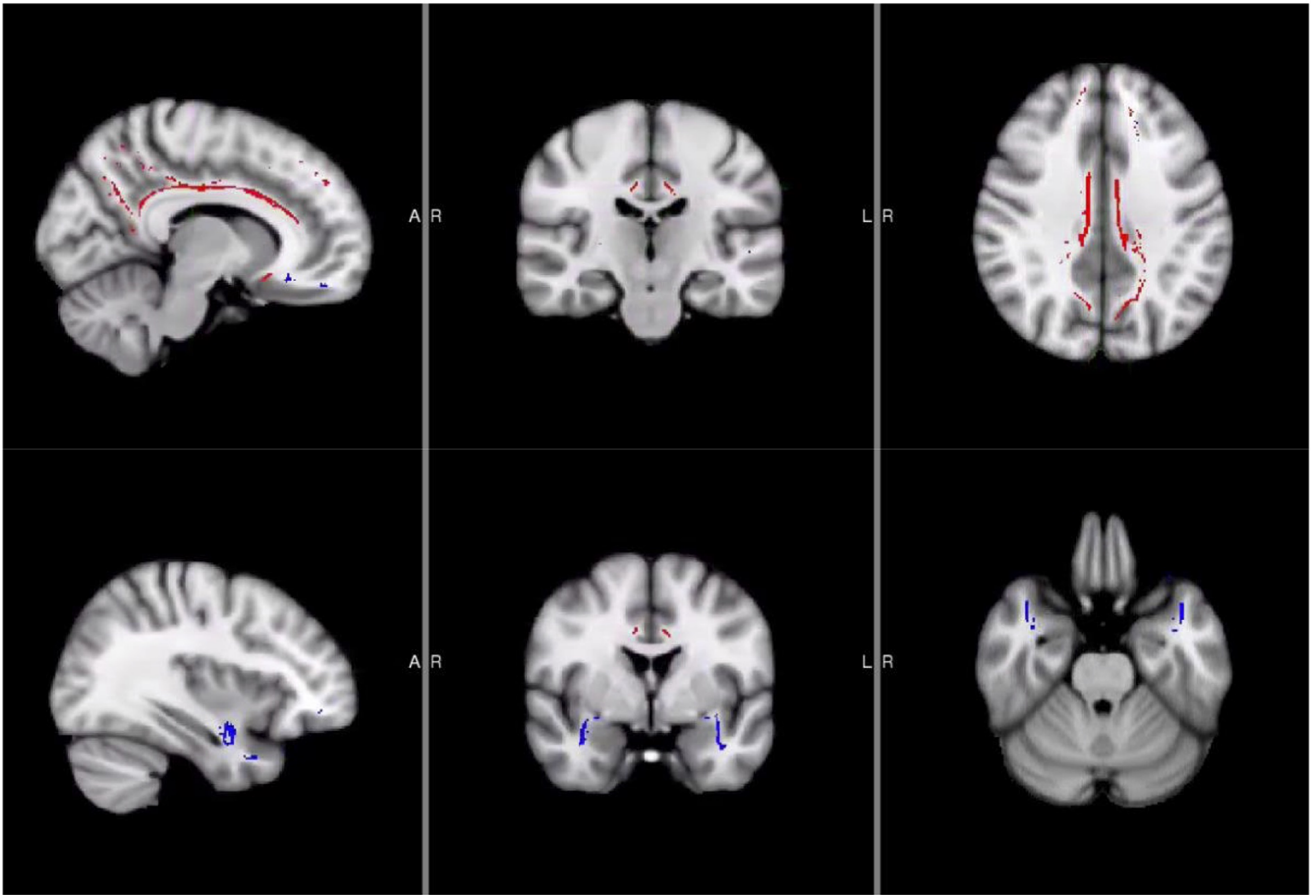
Locations of Uncinate Fasciculus and Cingulum Masks. Areas in red were used for the cingulum masks while blue areas were used for the uncinate fasciculus masks.

Using the steps for Tract-Based Spatial Statistics (TBSS) in FSL (38), we performed nonlinear registration of the GFA maps into standard space, creation of mean GFA images and a white matter “skeleton” for each individual. This was followed by a projection of the GFA data from all subjects onto the mean GFA skeleton. For the purpose of examining brain-behavior correlates within the NSSI group, the JHU-ICBM-tracts-maxprob-thr0-1 mm atlas was used to create region of interest (ROI) masks for the right and left cingulum and uncinate fasciculus, which are both tracts known to be critical for self-regulation. The ROI masks were multiplied with the GFA mean skeleton to restrict the analyses to voxels within the skeleton and in the tracks of interest (see **Figure 1**). Finally, *fslmeants* was used to extract average GFA values within the skeleton portion of each of the four ROIs for all participants for clinical correlations.

### Statistical Analysis

Group comparisons examining differences between NSSI and HC groups in GFA maps, while controlling for age, were completed using GLM modeling and the Threshold-Free Cluster Enhancement [TFCE; (39)] option with *p-*value < .01.

Within the NSSI group, we conducted correlations between GFA and clinical measures using partial Pearson’s correlations controlling for age and IQ. To allow our analyses to be more specific to NSSI, as opposed to depressive symptoms, we included BDI scores as covariates. We performed correlations between GFA of the right and left uncinate fasciculus and scores from the 7 DERS scales, 4 BIS scales, and the BOR-S scale from the PAI/PAI-A. Given the total of 24 comparisons and our hypothesis that higher scores on these clinical measures will be associated with lower GFA values, we used a one-tailed *p*-value < .002 as our level of significance. We used the same method for the right and left cingulum including using a one-tailed *p*-value < .002. We elected to use one-tailed *p*-values due to our *a priori* hypotheses that higher scores on clinical measures will correspond to lower GFA values.

We also explored whether there were any associations between NSSI severity (frequency and duration of NSSI) and GFA in the cingulum and uncinate fasciculus. We used a one-tailed *p*-value < .0125 in recognition of 4 comparisons for each NSSI frequency and duration.

## Results

### Demographic and Clinical Characteristics

Overall, 29 NSSI and 22 HC completed all study procedures. After one subject was excluded due to poor dMRI data quality, data from 28 NSSI and 22 HC participants were used for the final analyses. The number of NSSI participants who completed each of the clinical measures for this study varied. Further demographic and clinical characteristics for the sample can be found in **Table 1**.

**Table 1:** Participant demographics.

Demographic Characteristics	NSSI (n = 28)	Controls (n = 22)
Age (mean years ± SD)	17.53 ± 2.36	17.69 ± 2.26
IQ (mean ± SD)	105.78 ± 10.68 (n = 27)	110.05 ± 9.43 (n = 20)
Right Handed – n (%)^a^	24 (89%; n = 27)	19 (100%; n = 19)
***Ethnicity* – n (%)**^b^		
White	26 (93%)	19 (86%)
African American	1 (4%)	1 (5%)
Hispanic	3 (11%)	0
Asian	0	2 (7%)
Other	1 (4%)	0
***Clinical Measures***		
DERS Total**	119.17 ± 22.22 (n = 24)	60.63 ± 9.39 (n = 19)
DERS Awareness**	20.63 ± 6.35 (n = 24)	12.26 ± 3.35 (n = 19)
DERS Clarity**	16.69 ± 4.30 (n = 26)	8.48 ± 1.66 (n = 21)
DERS Goals*	17.92 ± 5.54 (n = 24)	12.21 ± 4.72 (n = 19)
DERS Impulse**	18.88 ± 5.91 (n = 24)	7.54 ± 2.03 (n = 19)
DERS Nonacceptance**	18.92 ± 6.21 (n = 24)	9.11 ± 2.89 (n = 19)
DERS Strategies**	26.29 ± 5.65 (n = 24)	10.95 ± 3.10 (n = 19)
BIS Total**	73.12 ± 10.47 (n = 26)	57.24 ± 8.24 (n = 21)
BIS Attentional**	21.00 ± 3.71 (n = 26)	15.05 ± 3.07 (n = 21)
BIS Motor**	24.31 ± 5.63 (n = 26)	18.76 ± 3.86 (n = 21)
BIS Nonplanning*	27.81 ± 3.67 (n = 26)	23.43 ± 4.91 (n = 21)
PAI/PAI-A Borderline-Self Harm Subscale**	64.88 ± 15.06 (n = 25)	44.95 ± 11.49 (n = 19)
***NSSI Characteristics***		
Age of first NSSI (mean ± SD)	11.96 ± 3.03 (n = 27)	
Lifetime Cutting Episodes (mean ± SD)	131.11 ± 195.43	
Estimated Cutting Episodes per Week (mean ± SD)^c^	0.75 ± 1.16	
Duration (years) of NSSI (mean ± SD)^d^	5.43 ± 3.85 (n = 27)	
***Current Diagnoses* – n (%)^e^**		
Major Depressive Disorder	16 (57%)	
Depressive Disorder NOS	5 (18%)	
Generalized Anxiety Disorder	8 (29%)	
Anxiety Disorder NOS	2 (7%)	
Social Phobia	1 (4%)	
Specific Phobia	3 (11%)	
Panic Disorder	3 (11%)	
Post-Traumatic Stress Disorder	5 (18%)	
Obsessive Compulsive Disorder	2 (7%)	
Eating Disorder NOS	1 (4%)	
ADHD	2 (7%)	
Alcohol Dependence	2 (7%)	
No Current Disorder	5 (18%)	
***Medications***		
Currently Medicated	12 (43%)	
Antidepressants	9 (32%)	
Stimulants	2 (7%)	
Antipsychotics	1 (4%)	
Antianxiety/Benzodiazepines	4 (14%)	
Other Psychotropics	1 (4%)	

*p < .005.

**p < .001.

a Post-hoc analyses indicated that differing handedness did not affect study findings.

b Participants were able to endorse more than one option for ethnicity.

c Consensus between ISAS and DSHI was calculated to determine average number of cutting episodes per week. These are pre-Winsorized scores.

d Duration of NSSI calculated by subtracting age of first NSSI from current age.

e Diagnoses include both primary and comorbid disorders.

### dMRI

**Figure 2:**
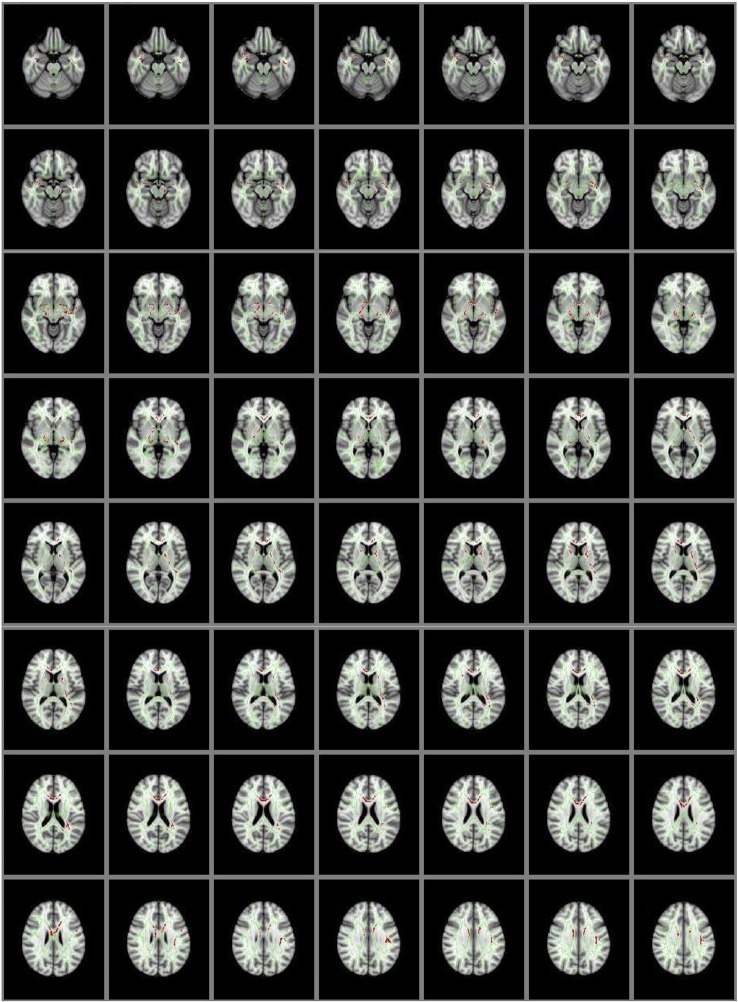
Group Differences in GFA: Controls > NSSI. White matter tracts in red show where controls have significantly greater GFA than NSSI. This is overlaid on the mean GFA skeleton (light green). Findings were significant at p < .01.

The whole brain group comparison analyses revealed several areas that showed significantly lower GFA in the NSSI group when compared to the HC group at a corrected *p* < .01 (corrected through permutation testing within TFCE as described in 39). In addition to the cingulum and uncinate fasciculus as predicted, these areas also included bilateral superior and inferior longitudinal fasciculi, anterior thalamic radiation, callosal body, and corticospinal tract. **Figure 2** depicts the locations of these group differences.

Lower GFA of the left and right uncinate fasciculus was associated with higher total scores on the attentional subscale of the BIS. A correlation matrix can be found in **Table 2**, which includes comparisons that were significant at an uncorrected *p* < .05. For the left and right cingulum, there were no significant associations with measures of self-regulation (DERS and BIS). A correlation matrix can be found in **Table 3**. Finally, lower GFA of the left cingulum was associated with a longer duration of NSSI. There were no significant correlations between other severity indices (e.g., average number of episodes) and GFA. These in addition to results from other comparisons can be found in the correlation matrix in **Table 4**.

**Table 2:** Self-regulation and uncinate fasciculus correlations.

Control Variables: Age & BDI & IQ		Correlations	
		Left Uncinate Fasciculus	Right Uncinate Fasciculus
BOR-S	Correlation	-.341	-.329
	Significance (1-tailed)	.051	.062
	df	21	21
BIS Attentional	Correlation	-.668	-.573
	Significance (1-tailed)	< .001*	.002*
	df	22	22
BIS Motor	Correlation	-.383	-.374
	Significance (1-tailed)	.032	.036
	df	22	22
BIS Nonplanning	Correlation	-.306	-.137
	Significance (1-tailed)	.073	.262
	df	22	22
BIS Total	Correlation	-.550	-.447
	Significance (1-tailed)	.003	.014
	df	22	22
DERS Nonaccept	Correlation	.109	.066
	Significance (1-tailed)	.315	.385
	df	20	20
DERS Goals	Correlation	-.348	-.300
	Significance (1-tailed)	.056	.088
	df	20	20
DERS Impulse	Correlation	-.038	-.066
	Significance (1-tailed)	.433	.386
	df	20	20
DERS Awareness	Correlation	.360	.119
	Significance (1-tailed)	.050	.298
	df	20	20
DERS Strategies	Correlation	.064	.074
	Significance (1-tailed)	.389	.371
	df	20	20
DERS Clarity	Correlation	.162	.020
	Significance (1-tailed)	.224	.463
	df	22	22
DERS Total	Correlation	.079	-.037
	Significance (1-tailed)	.363	.435
	df	20	20

*Meets criteria for significance based on corrected p-value < .002.

**Table 3:** Self-regulation and cingulum correlations.

Control Variables: Age & BDI & IQ		Correlations	
		Left Cingulum	Right Cingulum
BOR-S	Correlation	-.442	-.507
	Significance (1-tailed)	.017	.007
	df	21	21
BIS Attentional	Correlation	-.392	-.455
	Significance (1-tailed)	.029	.013
	df	22	22
BIS Motor	Correlation	-.411	-.502
	Significance (1-tailed)	.023	.006
	df	22	22
BIS Nonplanning	Correlation	-.223	-.333
	Significance (1-tailed)	.147	.056
	df	22	22
BIS Total	Correlation	-.436	-.547
	Significance (1-tailed)	.017	.003
	df	22	22
DERS Nonaccept	Correlation	.066	-.048
	Significance (1-tailed)	.384	.417
	df	20	20
DERS Goals	Correlation	.053	.019
	Significance (1-tailed)	.408	.467
	df	20	20
DERS Impulse	Correlation	-.330	-.268
	Significance (1-tailed)	.067	.114
	df	20	20
DERS Awareness	Correlation	-.179	-.033
	Significance (1-tailed)	.212	.332
	df	20	20
DERS Strategies	Correlation	.358	.279
	Significance (1-tailed)	.051	.104
	df	20	20
DERS Clarity	Correlation	-.277	-.235
	Significance (1-tailed)	.095	.135
	df	22	22
DERS Total	Correlation	-.076	-.077
	Significance (1-tailed)	.368	.367
	df	20	20

**Table 4:** Duration of non-suicidal self-injury (NSSI) and cutting frequency and GFA correlations.

Control Variables: Age & BDI & IQ		Correlations			
		Left Cingulum	Right Cingulum	Left Uncinate Fasciculus	Right Uncinate Fasciculus
Weekly Cutting Episodes	Correlation	.000	-.113	-.019	.005
	Significance (1-tailed)	.500	.296	.465	.491
	df	23	23	23	23
Duration of NSSI	Correlation	-.505	-.452	-.285	-.268
	Significance (1-tailed)	.005*	.012*	.084	.097
	df	23	23	23	23

*Meets criteria for significance based on corrected p-value < .0125.

## Discussion

In this study of white matter microstructure in adolescents and young adults with NSSI, we report extensive group differences in GFA between females with NSSI and healthy controls in the uncinate fasciculus, cingulum, and several other white matter tracts throughout the brain; which is consistent with our hypotheses. This suggests NSSI may be associated with a broad array of white matter disorganization that extends beyond the scope of the present study. However, future research investigating the potential functional meaning of these other white matter deficits may be beneficial. We also report associations between GFA in the uncinate fasciculus and cingulum and clinical characteristics (measures of self-regulation and NSSI characteristics). Specifically, higher levels of attentional impulsivity; characterized by racing thoughts, difficulty with focus, and intrusive thoughts (31); were associated with lower GFA in the left uncinate fasciculus. Additionally, longer duration of NSSI was associated with lower GFA in the left cingulum. These findings suggest that among participants with NSSI, greater psychopathology in key domains (impulsivity, severity of self-harm) may be explained by greater disorganization in key frontolimbic white matter tracts in this still-developing population.

### Group Differences in Whole Brain GFA Between NSSI and HC

Adolescents and young adults with NSSI showed lower GFA compared to the HC group in the uncinate fasciculus and cingulum, which is consistent with our hypotheses. In addition, the NSSI group showed lower GFA in several other areas including the inferior and superior longitudinal fasciculi, callosal body, forceps major and minor, anterior thalamic radiation, and corticospinal tract. To our knowledge, only one other study has examined differences in structural connectivity between those with and without self-injury and found compromised white matter microstructure within the frontal lobe in adults with BPD (15). The present study differs from this previous study as it examines adolescents, examines NSSI across diagnoses, uses a larger sample size, employs methods that result in a potentially more accurate scalar measure of white matter integrity (GFA), and also investigates NSSI more explicitly as it is unclear whether the previous study included suicidal self-injury.

### ROI-Specific GFA Associations With Clinical Measures

We also examined the association between clinical measures and GFA within the NSSI group while controlling for age, IQ, and BDI-II scores. Lower GFA within the uncinate fasciculus, which serves brain regions implicated in self-regulation, was significantly associated with higher scores on the attentional subscale of the BIS. Given the role the uncinate fasciculus plays in serving as a connection between subcortical structures and frontal regulatory regions, the present findings suggest that those who experience racing and intrusive thoughts or difficulties with focusing on tasks may show compromised white matter organization within this tract. Decreased FA in the uncinate fasciculus has been associated with BPD (40, 41), emotion dysregulation disorders (42), and suicide attempts (43–46). While the relationship between NSSI and impulsivity remains controversial, a recent review provides helpful insight regarding the complexities of this relationship. Lockwood and colleagues (17) highlight that mood-dependent impulsivity, such as that measured by Negative Urgency in the UPPS Impulsive Behavior Scale (47), predisposes an individual to begin engaging in NSSI; while higher scores on more cognitively-related facets of impulsivity were more reflective of recent NSSI, and thus may serve to maintain the behavior. This is consistent with the relationship between BIS scores and GFA within our sample of NSSI participants as they had been engaging in recent self-injury. However, it is necessary to further elaborate on these relationships by also incorporating measures investigating mood-dependent impulsivity.

We also found that longer duration of NSSI was associated with lower GFA within the left and right cingulum. Because we controlled for current age in these analyses, this finding suggests that the impaired white matter integrity of this region among those with NSSI may be the result of a cumulative effect over time. However, it is also important to consider the high likelihood that any existing psychopathology had developed concurrently, or had already existed, around the time of first NSSI episode. Although some studies have reported null findings regarding differences in FA of the cingulum between psychiatric samples and controls (48, 49), a meta-analysis of adolescents with major depressive disorder (MDD) found that overall, those with depression had decreased FA within this region (50). Our finding highlights the importance of early intervention and the utility it may have in preventing aberrant, or restoring normal, neurodevelopmental trajectories. Further, given the number of functions in which the cingulum plays a role, including emotion processing, pain, and executive functioning (18), it is imperative that there is continued investigation into this possible disruption as it may lead to entrenchment of maladaptive behaviors and poorer prognosis.

### Strengths and Limitations

This study represents a significant advancement of existing NSSI work as it used an approach to the dMRI data that lessens the impact of crossing fibers when compared to dMRI methods used in previous studies. Unlike many previous studies of NSSI, which investigate the behavior in the context of a specific diagnosis, the present study examines the neural circuitry of NSSI across diagnoses. As a strength, the presence of varying types and levels of psychopathology seen in this study is consistent with what has been found in larger studies of NSSI (51, 52) and may reflect a more representative sample of those with NSSI. However, taking a diagnostic-independent approach also poses a limitation as it is difficult to determine whether findings are specific to NSSI or to psychopathology more broadly. We aimed to limit this influence by controlling for depression symptoms when performing our within-NSSI group analyses, as depressive disorders were the most common diagnoses in our sample. However, it would be beneficial for future research to incorporate a psychiatric control group that is matched to the NSSI group on diagnosis and severity level/level of impairment. Considerations of external validity should be considered given that the present study consisted of only females and primarily older adolescents/young adults. Further, given that there was an experimental intervention offered as part of the larger study, participants may be more likely to be willing to disclose NSSI and be treatment-seeking. Additional limitations include the inability to generalize to males, the likelihood that the present sample was ready to seek treatment, and the sample consisting of mainly older adolescents.

The cross-sectional design of this study is also a limitation, particularly when interpreting the association between longer duration of NSSI and lower GFA. While we do believe this finding supports a treatment approach with an earlier intervention and prevention, it is still imperative to fully explore whether this white matter disruption was present before or after NSSI onset. Longitudinal designs may also help in developing our understanding of the mechanisms of change with successful intervention strategies, which may then be used to target neurobiologically-based deficits associated with NSSI.

It is possible that these white matter anomalies are common across a range of psychopathology. Relying on FA as opposed to GFA, studies examining dMRI have found compromised white matter microstructure associated with psychopathology within these tracts including adults with MDD (53), PTSD (54), and childhood adversity (55), and adolescents with BPD (41). Additionally, a meta-analysis of FA in emotional disorders (MDD, bipolar disorder, social anxiety disorder, obsessive-compulsive disorder, and PTSD) found significantly lower FA compared to healthy controls in the forceps minor, uncinate fasciculus, anterior thalamic radiation, and superior longitudinal fasciculus (56). Given that most individuals in the NSSI group in the present study had a current diagnosis of emotional disorders, such as those included in the meta-analysis by Jenkins and colleagues (56), the widespread findings of lower GFA in this group may reflect overall psychopathology present in this sample. With this in mind, future research examining GFA in adolescents with NSSI should consider incorporating a psychiatric control group to allow for greater specificity in understanding the aberrations that are unique to NSSI.

Finally, our sample size, while much larger than the previous study examining self-injury and white matter integrity, limits our ability to conduct correlational analyses given the number of comparisons and limited power. While there are other clinical measures that may be of interest to explore within this sample, such as identity disturbance and interpersonal sensitivity, we limited our analyses to these specific constructs of self-regulation given the existing literature in NSSI. In the future, larger studies will be better suited to more fully examine other constructs implicated in NSSI.

## Conclusion

This is among one of the first studies to provide evidence for the role of compromised white matter organization and its relationship to clinical measures among adolescents and young adults with NSSI. Categorical analyses revealed that compared to healthy controls, the NSSI group exhibited widespread white matter disruption, consistent with other forms of psychopathology including depression and suicide. Dimensional analyses revealed that among those with NSSI, levels of attentional impulsivity and the duration of NSSI within the NSSI group were associated with lower white matter integrity in the uncinate fasciculus and cingulum. Clinically, these findings provide some insight as to how interventions that focus on self-regulation, such as dialectical behavior therapy (DBT), have shown success in treating NSSI (57). Additionally, the association between duration of NSSI and GFA provides further support for the importance of early intervention in hopes to restore healthy neurodevelopmental trajectories. However, longitudinal research is needed to understand when and how these white matter abnormalities develop, whether they predispose adolescents to developing maladaptive behaviors such as NSSI or if they emerge later in the disease course. This knowledge may then contribute to the foundation of more effective and targeted interventions.

## Data Availability Statement

The datasets generated for this study are available on request to the corresponding author.

## Ethics Statement

The studies involving human participants were reviewed and approved by University of Minnesota Institutional Review Board. Written informed consent to participate in this study was provided by the participants’ legal guardian/next of kin.

## Author Contributions

MW conceived and designed the analyses, collected the data, performed analyses, and wrote the manuscript. BM conceived and designed the analyses, collected the data, performed analyses, and contributed to manuscript writing. BK-D assisted in study conception and design, data collection, and contributed to manuscript writing. EB contributed to manuscript writing and data collection. MF contributed biostatistical guidance. DH contributed to manuscript writing. KL contributed to study conception and design. KC contributed funding, conception and design, data collection, analyses, and manuscript writing.

## Funding

This study was funded by the National Institute of Mental Health grant 1R21MH094558 (KC), the University of Minnesota Academic Health Center Faculty Research Development Grant Program #11.12, the University of Minnesota Clinical and Translational Science Award UL1TR000114, and the National Institute of Biomedical Imaging and Bioengineering P41 EB015894 granted to the Center for Magnetic Resonance Research. MW’s time on this study was funded by the University of Minnesota’s Doctoral Dissertation Fellowship.

## Conflict of Interest

The authors declare that the research was conducted in the absence of any commercial or financial relationships that could be construed as a potential conflict of interest.
