# Corrigendum: White Matter Microstructure in Adolescents and Young Adults With Non-Suicidal Self-Injury

**DOI:** 10.3389/fpsyt.2020.00157

**Published:** 2020-03-05

**Authors:** Melinda Westlund Schreiner, Bryon A. Mueller, Bonnie Klimes-Dougan, Erin D. Begnel, Mark Fiecas, Dawson Hill, Kelvin O. Lim, Kathryn R. Cullen

**Affiliations:** ^1^Department of Psychiatry, University of Utah, School of Medicine, Salt Lake City, UT, United States; ^2^Department of Psychiatry and Behavioral Sciences, University of Minnesota Medical School, Minneapolis, MN, United States; ^3^Department of Psychology, University of Minnesota College of Liberal Arts, Minneapolis, MN, United States; ^4^Division of Biostatistics, University of Minnesota School of Public Health, Minneapolis, MN, United States

**Keywords:** non-suicidal self-injury, neuroimaging, fractional anisotropy, uncinate fasciculus, cingulum, adolescents

An author's name was incorrectly spelled as “Mark Fiecus”. The correct spelling is “Mark Fiecas”.

The authors apologize for this error and state that this does not change the scientific conclusions of the article in any way. The original article has been updated.

